# Active zone proteins are transported via distinct mechanisms regulated by Par-1 kinase

**DOI:** 10.1371/journal.pgen.1006621

**Published:** 2017-02-21

**Authors:** Kara R. Barber, Julia Tanquary, Keegan Bush, Amanda Shaw, Michael Woodson, Michael Sherman, Yogesh P. Wairkar

**Affiliations:** 1 George and Cynthia Mitchell Center for Neurodegenerative Diseases, Department of Neurology, University of Texas Medical Branch, Galveston, TX, United States of America; 2 Neuroscience Graduate Program, Department of Neurology, University of Texas Medical Branch, Galveston, TX, United States of America; 3 Summer Undergraduate Research Program, UTMB, Department of Neurology, University of Texas Medical Branch, Galveston, TX, United States of America; 4 Sealy Center for Structural Biology, UTMB, Department of Biochemistry and Molecular Biology, University of Texas Medical Branch, Galveston, TX, United States of America; Stanford University School of Medicine, UNITED STATES

## Abstract

Disruption of synapses underlies a plethora of neurodevelopmental and neurodegenerative disease. Presynaptic specialization called the active zone plays a critical role in the communication with postsynaptic neuron. While the role of many proteins at the active zones in synaptic communication is relatively well studied, very little is known about how these proteins are transported to the synapses. For example, are there distinct mechanisms for the transport of active zone components or are they all transported in the same transport vesicle? Is active zone protein transport regulated? In this report we show that overexpression of Par-1/MARK kinase, a protein whose misregulation has been implicated in Autism spectrum disorders (ASDs) and neurodegenerative disorders, lead to a specific block in the transport of an active zone protein component- Bruchpilot at *Drosophila* neuromuscular junctions. Consistent with a block in axonal transport, we find a decrease in number of active zones and reduced neurotransmission in flies overexpressing Par-1 kinase. Interestingly, we find that Par-1 acts independently of Tau-one of the most well studied substrates of Par-1, revealing a presynaptic function for Par-1 that is independent of Tau. Thus, our study strongly suggests that there are distinct mechanisms that transport components of active zones and that they are tightly regulated.

## Author summary

Synapses consist of pre- and postsynaptic partners. Proper function of active zones, a presynaptic component of synapse, is essential for efficacious neuronal communication. Disruption of neuronal communication is an early sign of both neurodevelopmental as well as neurodegenerative diseases. Since proteins that reside in active zones are used so frequently during the neuronal communication, they must be constantly replenished to maintain active zones. Axonal transport of these proteins plays an important role in replenishing these vital components necessary for the health of active zones. However, the mechanisms that transport components of active zones are not well understood. Our data suggest that there are distinct mechanisms that transport various active zone cargoes and this process is likely regulated by kinases. Further, our data show that disruption in the transport of one such active zone components causes reduced neuronal communication emphasizing the importance of the process of axonal transport of active zone protein(s) for neuronal communication. Understanding the processes that govern the axonal transport of active zone components will help dissect the initial stages of pathogenesis in both neurodevelopmental and neurodegenerative diseases.

## Introduction

Effective communication between neurons is maintained by synapses via their pre- and postsynaptic specializations called active zones and postsynaptic densities respectively. Active zones are composed of many proteins that are important for the efficient release of synaptic vesicles- a pre-requisite for efficacious neuronal communication[1, 2]. Proteins present at the active zones form an important presynaptic network for the regulation of vesicle release at all chemical synapses. Indeed, many proteins that regulate synapses are disrupted in both neurodevelopmental as well as neurodegenerative diseases[3–5]. One such protein, microtubule associated regulatory protein (MARK)/ partitioning-defective 1 (Par-1) is implicated in both neurodevelopmental [6–8]and neurodegenerative diseases[9–12] but the mechanisms by which it disrupts synapses is unclear.

MARK1 levels are elevated in Autism spectrum disorders (ASDs), a neurodevelopmental disorder[6]. Interestingly, MARK1 is overexpressed specifically in the prefrontal cortex- a region highly implicated in ASDs [6]. On the other hand, MARK4 is overexpressed in neurodegenerative diseases and is thought to hyperphosphorylate Tau [13, 14]. Indeed, the site that is phosphorylated by the MARK/Par-1 is hyperphosphorylated in post-mortem brains of patients with frontotemporal dementia (FTD)[11]. Thus, elevated levels or activity of MARK/Par-1 is implicated in both neurodevelopmental and neurodegenerative diseases. While there is good evidence for the role of MARK/Par-1 in regulating postsynaptic density during development[15], it is unclear whether it has any presynaptic role.

In this study, we show for the first time that presynaptic overexpression of Par-1 regulates the axonal transport of an active zone protein- Bruchpilot (BRP). Decreased axonal transport of BRP due to presynaptic overexpression of Par-1 lead to a significant decrease in the number of BRP marked active zones at the synaptic terminals. Furthermore, consistent with a decrease in BRP protein at the synapse[16], ultrastructural analysis demonstrated a decrease in the number of dense bars and deficits in synaptic transmission. Finally, our data show that MARK/Par-1 affects the axonal transport of BRP independent of endogenous *Drosophila* Tau (dTau), implicating that a novel substrate of MARK/Par-1 mediates the axonal transport of BRP. Together, these data suggest that different components of active zones are transported separately by distinct mechanisms, and that these processes are likely to be tightly regulated by kinases.

## Results

### Overexpression of Par-1 in the presynaptic neurons leads to specific accumulation of BRP in axons

**Fig 1:**
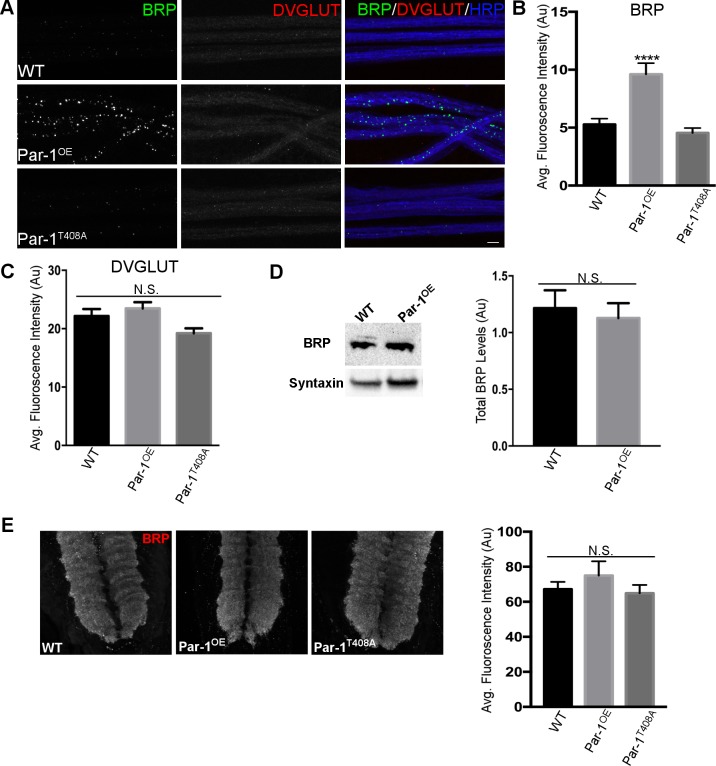
**A**) Representative confocal image stacks showing axons from WT, presynaptic overexpression of Par-1 (Par-1^OE^) and inactive Par-1 (Par-1^T408A^) using BG380-Gal4. Third instar larvae were stained with antibodies against BRP (Green), DVGLUT (Red) and HRP (Blue). Scale bar = 10μm **B**) Quantification of BRP intensity from axons in A. n = 12, **** = p<0.0001. **C**) Quantification of DVGLUT intensity from axons in A. n = 12, p = 0.12. **D**) Representative Western blots and bar graphs showing quantification of BRP levels in WT and Par-1^OE^ brains. Syntaxin was used as a loading control. N = 3 p = 0.7124. **E**) Representative images and bar graphs showing quantification of VNCs stained with BRP (Red) from identical genotypes as in 1A. N = 12 p = 0.46. Scale bar = 10μm. Error bars represent S.E.M.

Increase in levels or activity of Par-1/MARK is associated with both neurodevelopmental disorders like ASD[6–8] and neurodegenerative disorders like FTD[11]. Since synapse is the common underlying unit disrupted in both these disorders[3–5] we wanted to test the effects of elevated levels of presynaptic Par-1 on synapses. To test this, we overexpressed Par-1 presynaptically using the UAS-GAL4 binary system[17]. BG380-GAL4[18] driver was used to overexpress of Par-1 (Par-1^OE^) specifically in presynaptic neurons. Neuromuscular Junction (NMJ) preparations were then stained with antibodies against the active zone marker (Bruchpilot (BRP), [16]), synaptic vesicle marker (DVGLUT[19]) and neuronal membrane marker (Horse Radish Peroxidase(HRP)[20]) to visualize synapses. Overexpression of Par-1 in presynaptic neurons resulted in significant accumulation of BRP in axons (Fig 1A and 1B). This was observed using multiple presynaptic drivers (S1 Fig). While all the tested presynaptic drivers showed qualitatively similar increases in accumulation of BRP in axons, driving the same transgene postsynaptically using postsynaptic driver G7-Gal4[21] did not result in the accumulation of BRP within axons (S1 Fig), suggesting that this was a cell-autonomous effect.

Surprisingly, DVGLUT or HRP did not accumulate within the axons (Fig 1A and 1C), indicating that overexpression of Par-1 may result in specific accumulation of only a subset of synaptic cargo in the axons. Importantly, overexpression of inactive Par-1 (Par-1^T408A^, [22]) did not result in accumulation of BRP within axons (Fig 1A and 1B), indicating that BRP accumulations were unlikely to be an unintended consequence of overexpression of Par-1 kinase.

**Fig 2:**
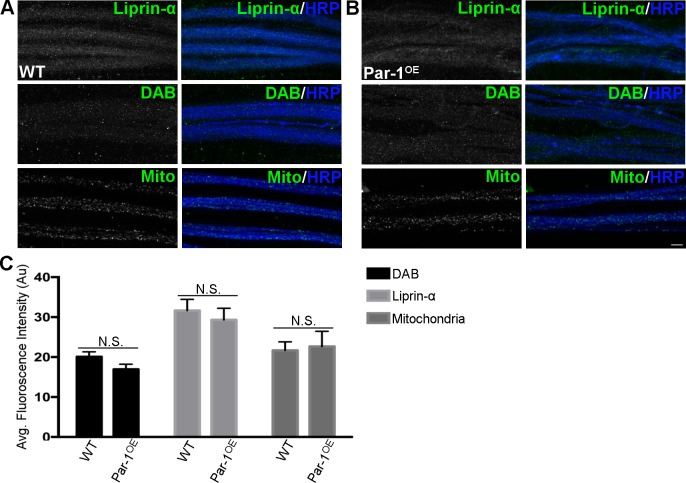
**A**) Representative confocal image stacks showing axons from WT and **B**) Par-1^OE^ third instar larvae stained against Liprin-α (Green), DAB (Green), mito-GFP (Green) and HRP (Blue). Scale bar = 10μm. **C**) Quantification of Liprin-α, DAB, mito-GFP intensities from axons. N = 12, Liprin-α p = 0.49, DAB p = 0.09, and mito-GFP p = 0.82. Error bars represent S.E.M.

To further test our hypothesis that overexpression of Par-1 may lead to a specific axonal transport defect of BRP; we labeled the axons using markers of various cargoes that are transported within the axons. We used the following markers: Liprin-α (another marker of active zones,[23]), and disabled (DAB, a marker for endocytic zones,[24]). The levels of Liprin-α and DAB in the axons of flies overexpressing Par-1 were similar to the levels of these proteins in WT flies (Fig 2A–2C), providing further evidence that overexpression of Par-1 results in specific accumulation of BRP in axons. Next, we tested the transport of mitochondria, which is mediated by Milton and Miro[25]. To test this, we generated flies that express mito-GFP in the presynaptic neurons along with overexpression of Par-1. To account for the possible “dilution” of GAL4 due to two UAS promoters (UAS-mito-GFP and UAS-Par-1^OE^), we generated flies that carry UAS-GFP and UAS-Par-1 as a control. Expression of mito-GFP in wild type flies showed many mitochondria within the axons. Consistent with our hypothesis, flies overexpressing mito-GFP in Par-1 overexpression background showed no significant changes in the levels (Fig 2A, 2B and 2C) or size (S3 Fig) of mitochondria within the axons while still showing accumulations of BRP, indicating that overexpression of Par-1 does not affect mitochondrial transport.

Finally, to test the possibility that increased transcription of BRP may lead to accumulation of BRP in axons[26], we compared the BRP protein levels in the ventral nerve cords (VNC) between WT, Par-1^OE^ and Par-1^T408A^ flies. No significant differences were noted between the levels of BRP protein in the VNCs of these genotypes (Fig 1E). To confirm these data we also performed western blots using anti-BRP antibody on WT and Par-1^OE^ flies and did not observe any significant difference in the levels of BRP protein (Fig 1D). These data indicate that increased accumulations of BRP in axons of flies overexpressing Par-1 are unlikely to be due to increased levels of BRP protein. Taken together, our results strongly suggest that overexpression of Par-1 specifically affects the transport of BRP in the axons.

### Overexpression of Par-1 results in reduced T-bars and impaired synaptic transmission

**Fig 3:**
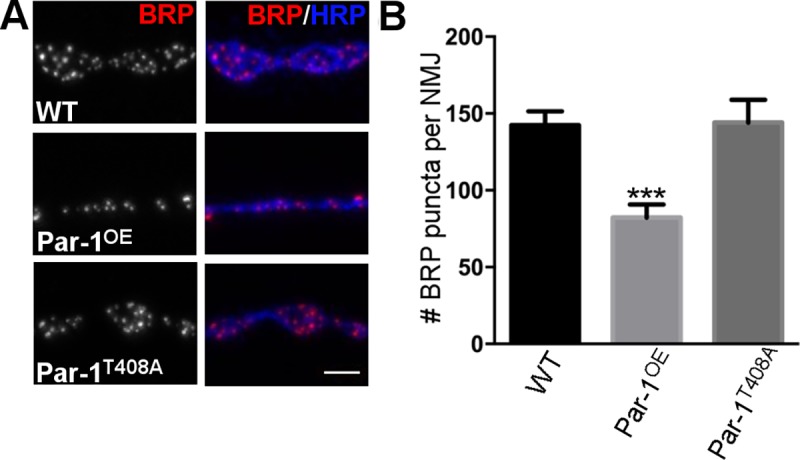
**A**) Representative images from WT, Par-1^OE^ and Par-1^T408A^ showing the NMJ synapses labeled with anti-BRP (Red) and anti-HRP (Blue) antibodies **B**) Quantification of BRP puncta per NMJ. N = 10, *** = p≤0.0001. Scale bar = 10μm. Error bars represent S.E.M.

To test whether block in axonal transport of BRP would lead to decreased levels of BRP at the synapses, we labeled the NMJ synapses with BRP[16] and HRP. Although we expected to see only a reduction in BRP at the synaptic terminals, we were surprised to find that there were some interesting differences between WT synapses and those overexpressing Par-1. First, as expected, there were significant reductions in the number of active zones marked by BRP (Fig 3A and 3B). Second, while the synaptic span was not significantly different in the flies overexpressing Par-1, the size of synaptic boutons was significantly reduced (S2 Fig). However, these changes did not affect the apposition of synapses quantified using number of BRP puncta apposed to DGluRIII patches, a marker of postsynaptic density (S2 Fig)[27]. These data show that although overexpression of Par-1 may cause specific defects in BRP transport, these defects may possibly lead to other changes at the synapse. It is not clear whether all of these changes are caused due to a block in axonal transport of BRP but levels of other synaptic proteins like Liprin-α and DAB are unaffected in flies overexpressing Par-1 (S3 Fig). We also compared the ratio of Liprin-α, DAB and BRP between the axons and the synapses. We found that the ratio was unchanged in Liprin-α and DAB. However, as expected, we found a significant increase in the ratio of BRP at axons versus synapses (S3 Fig). Interestingly, overexpression of Par-1^T408A^ does not show an increase in BRP within the axons or a reduction in BRP at the synapse arguing that these effects are not merely a consequence of overexpression of Par-1 (S2 Fig).

**Fig 4:**
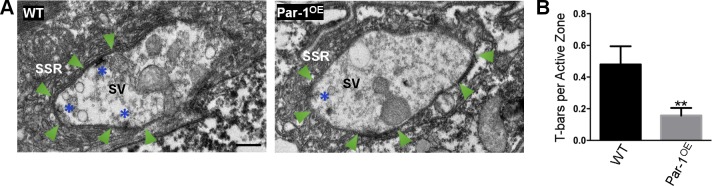
**A**) Representative electron micrographs from WT and Par-1^OE^ third instar larvae showing electron dense active zones (Arrows), T-bars (Asterisks), synaptic vesicles (SV) and sub-synaptic reticulum (SSR) in a single synaptic bouton. **B**) Quantification of T-bars from WT and Par-1^OE^ larvae. N = 17, ** = p = 0.0048. Scale Bar = 0.5μm. Error bars represent S.E.M.

To confirm our light-level findings, we performed ultrastructural studies at WT and Par-1^OE^ synapses. Consistent with previous reports showing that a reduction in BRP causes a decrease in number of T-bars[16, 28], we found a significant decrease in the total number of T-bars per active zones at the synapses of flies overexpressing Par-1 as compared to WT (Fig 4A and 4B). Taken together, our data so far demonstrate that overexpression of Par-1 in neurons leads to a specific block in axonal transport of BRP, which is the likely caused due to the reduction in T-bars at synapses.

**Fig 5:**
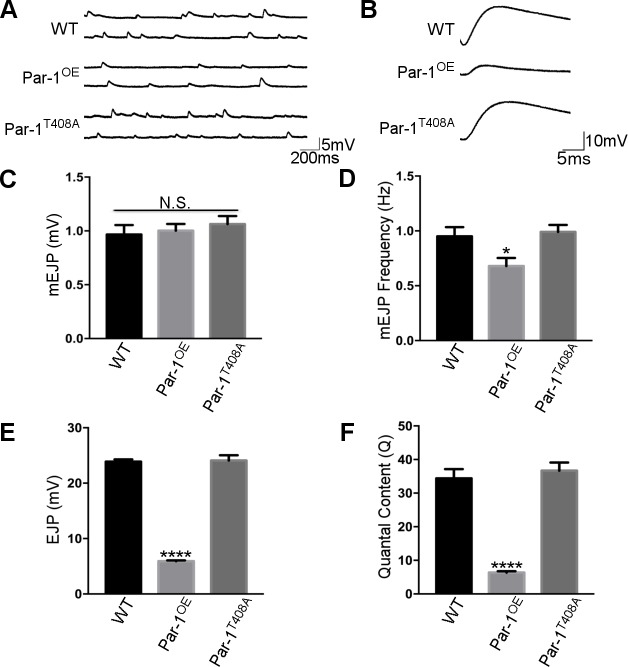
**A**) Representative mEJPs from WT, Par-1^OE^, and Par-1^T408A^. **B**) Representative EJPs from WT, Par-1^OE^ and Par-1^T408A^. **C**) Quantification of mEJP amplitude. N = 10, p = 0.65. **D**) Quantification of frequency of mEJPs N = 10, * = p<0.05. **E**) Quantification of EJP amplitude. N = 10, **** = p<0.0001. **F**) Quantification of Quantal Content. N = 10, **** = p<0.0001. Error bars represent S.E.M.

To test whether these synaptic changes result in defects in neurotransmission, we performed intracellular electrophysiological recordings from WT, Par-1^OE^ and Par-1^T408A^ flies. We did not observe any change in the amplitude (Fig 5A and 5C) of mini excitatory junction potentials (mEJPs), suggesting that the postsynaptic apparatus was unperturbed in Par-1^OE^ flies. However, the frequency of mEJPs was significantly reduced consistent with the decrease in number of release sites marked by BRP in Par-1^OE^ (Fig 5A and 5D). Furthermore, there was a dramatic reduction in the excitatory junction potential (EJP) amplitude in Par-1^OE^ flies (Fig 5B and 5E) pointing to a presynaptic defect. Calculation of the quantal content (EJP amplitude/mEJP amplitude) [29] showed a decrease in quantal content (Fig 5F) in flies overexpressing Par-1. These data are consistent with presynaptic deficits and are likely a consequence of fewer T-Bars[16, 28] and reduced size of synaptic boutons. Taken together, our data suggest that presynaptic elevation in the levels of Par-1 has both structural and functional consequences for the synapse.

### dTau does not mediate the specific transport of BRP

**Fig 6:**
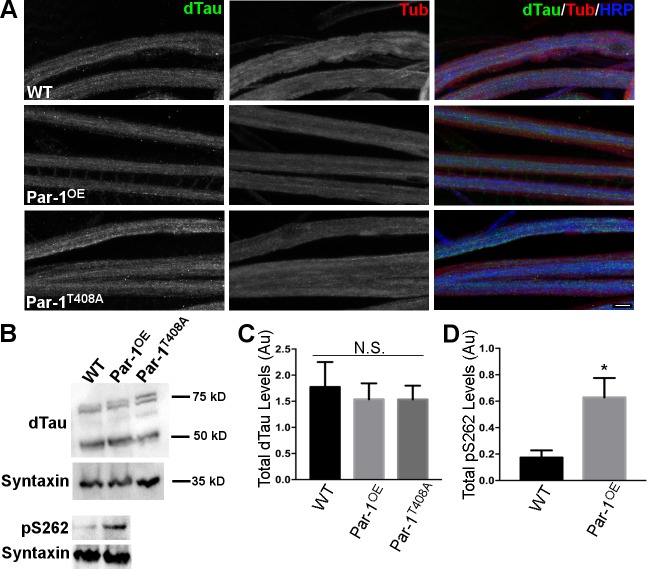
**A**) Representative confocal image stack showing axons of WT, Par-1^OE^, and Par-1^T408A^ third instar larvae stained with anti-dTau (Green), anti-Tubulin (Tub) (red) and anti-HRP antibodies (Blue). N = 10. Scale bar = 10μm. **B**) Representative Western blots from WT, Par-1^OE^ and Par-1^T408A^ using anti-dTau antibodies (upper panel). Western blot showing Tau phosphorylation at S262 site (pS262) of WT and Par-1^OE^ is shown in the bottom panel. Syntaxin was used as a loading control. **C**) Quantification of Western blots showing total dTau levels in the head lysates. N = 3 independent experiments, p = 0.87. Error bars represent S.E.M. **D**) Quantification of Western blots showing levels of pS262 levels of Tau. N = 3 independent experiments, * = p = 0.04. Error bars represent S.E.M.

Microtubules play an important role in axonal transport of synaptic cargo[30]. MARK/Par-1 kinase phosphorylates Tau[31]-a microtubule associated protein that binds and helps stabilize microtubules. Phosphorylation of Tau has been postulated to lead to its detachment from the microtubules leading to their destabilization[32, 33]. Thus, overexpression of Par-1 is expected to hyperphosphorylate Tau and lead to its detachment from microtubules making them unstable. To begin testing these possibilities, we first tested the levels of dTau using Western blot analysis on protein extracts from the ventral nerve cords (VNCs) of WT, Par-1^OE^ and Par-1^T408A^ flies using two previously characterized antibodies[34, 35]. The levels of dTau were not significantly different between these genotypes (Fig 6B and 6C), suggesting that Par-1 overexpression does not alter the levels of Tau in neurons. This raises the possibility that overexpressed Par-1 does not localize to the microtubules and is therefore unable to phosphorylate it. To test this possibility, we stained the axons of Par-1^OE^ flies with anti-Par-1 antibodies and compared its localization in WT axons. In Par-1^OE^ flies, Par-1 localized prominently within axons along with microtubules (S4 Fig) indicating that Par-1 localization to the microtubules was not hampered. We also tested the possibility that Par-1^T408A^ may not localize to axons and therefore would not phosphorylate its substrate (Tau). However, Par-1^T408A^ localized similar to that of overexpressed wild type Par-1 (S4 Fig). These experiments suggest that activity of Par-1 kinase is important to affect the transport of BRP within the axons.

**Fig 7:**
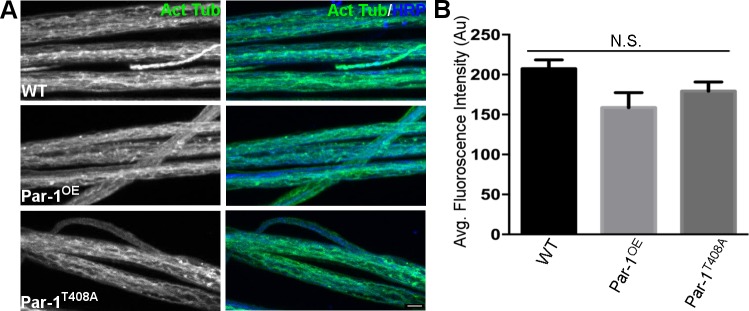
**A**) Representative images from WT, Par-1^OE^, and Par-1^T408A^ flies showing axons stained against the marker for stable microtubules- acetylated tubulin (Green) and HRP (Blue). **B**) Quantification of acetylated tubulin (Green) intensity in axons. N = 12, p = 0.064. Scale bar = 10μm.

Having established that overexpressed Par-1 can localize to the microtubules, we next wanted to test whether excess phosphorylation of Tau might cause its detachment from microtubules rendering them unstable[32, 33]. To test this, we first confirmed that overexpression of Par-1 could phosphorylate endogenous dTau. For this, we used an antibody that specifically recognizes the phospho-Ser262 on Tau (pS262) that is phosphorylated by Par-1[36]. We found that overexpression of Par-1 leads to an increase in dTau phosphorylation at Ser262 site (Fig 6B and 6D). We then stained the axons of WT, Par-1^OE^, and Par-1^T408A^ flies using the marker for stable microtubules-acetylated tubulin[37]. Distribution and levels of acetylated tubulin were unchanged in Par-1 overexpressing flies as compared to WT (Fig 7A and 7B), indicating that microtubule stability was uncompromised in flies overexpressing Par-1. Finally, we wanted to test whether dTau was mislocalized because of overexpression of Par-1. To test this we used the previously generated anti-dTau antibody[34] but because this antibody has not been used for staining axons or synapses, we first tested its specificity. For this, we performed co-localization experiments with overexpressed tau^GFP^ (S5 Fig). We found that overexpressed Tau^GFP^ co-localizes with anti-Tau antibody in axons. Furthermore, *dtau*^ko^[38] larval axons did not show any specific Tau staining within axons, (S5 Fig) demonstrating the specificity of anti-dTau antibody. Next, to test whether dTau localizes to microtubules we performed co-localization experiments of dTau with Tubulin-a marker for microtubules (Fig 6A). As expected, dTau and Tubulin co-localized in the axons indicating that dTau localizes to the microtubules in the axons.

We then tested whether dTau was mislocalized because of overexpression of Par-1 but did not find any evidence of mislocalization of dTau in flies overexpressing Par-1 (Fig 6A) within the axons. We also double labeled the axons for Tubulin and dTau to ascertain that dTau was still localized to the microtubules in flies overexpressing Par-1 (Fig 6A). These experiments suggest that instability of MT due to hyperphosphorylation of Tau is an unlikely reason for accumulations of BRP in axons of Par-1^OE^ flies.

Having shown that the levels of endogenous Tau were not altered in flies overexpressing Par-1, and that stability of microtubules was uncompromised, we wondered whether overexpression of Tau, which has been shown to cause neurodegeneration[39, 40], might have an effect on axonal transport of BRP. To test this possibility, we overexpressed tau^GFP^ in the presynaptic neurons. First, to confirm that dTau was overexpressed, we stained the axons with the anti-GFP antibody[41] (S5 Fig) and found that dTau was overexpressed. We then stained the axons of tau^GFP^ flies with antibodies against BRP to test whether dTau overexpression affected the axonal transport of BRP. We did not observe any significant difference in the levels or size of BRP puncta within the axons (S6 Fig) in flies overexpressing dTau as compared to WT. These data show that dTau overexpression does not cause accumulation of BRP within the axons.

**Fig 8:**
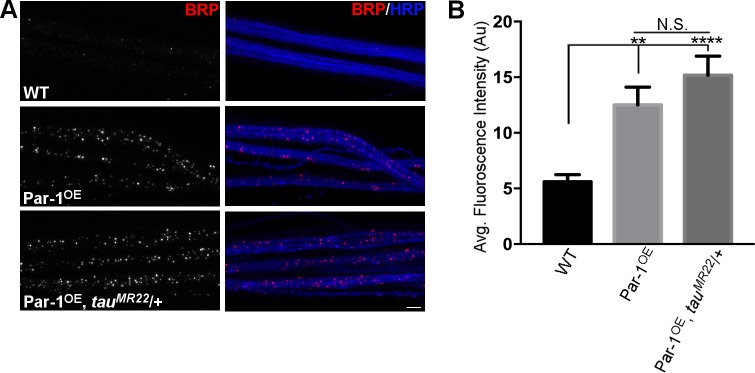
**A**) Representative third instar larvae stained with antibodies against anti-BRP (Red) and anti-HRP (Blue) from the following genotypes: WT, Par-1^OE^, Par-1^OE^, *tau*^*MR22*^/+. **B**) Quantification of BRP intensity in the axons from the genotypes. N = 15, ** = p<0.001, **** = p<0.0001. Scale bar = 10μm. Error bars represent S.E.M.

Finally, to test whether dTau may not be the endogenous substrate of Par-1 that mediates axonal transport of BRP, we generated a fly that overexpressed Par-1 in a *dtau* transheterozygote (Df(3R)tauMR22/+, [35]) (Par-1^OE^, tau^MR22^/+) because *tau*^*MR22*^ mutants are embryonic lethal[34, 35]. To confirm that *tau*^*MR22*^ heterozygotes had at least a 50% decrease because of deletion of one copy of dTau, we stained the *tau*^*MR22*^ heterozygous larvae with anti-dTau antibody. Levels of dTau in axons were reduced by ~70% (S7 Fig) in *tau*^*MR22*^ heterozygotes. If dTau were to mediate the effects of Par-1 overexpression on the axonal transport of BRP, we expect to see at least a partial suppression of BRP accumulations within the axons of flies that have reduced dTau levels. To test this, we stained WT, Par-1^OE^ and Par-1^OE^, *tau*^*MR22*^/+ fly NMJs with antibodies against BRP. As expected, Par-1^OE^ showed elevated levels of BRP in axons as compared to WT (Fig 8A and 8B). However, the levels of BRP protein in the axons of Par-1^OE^ and *tau*^*MR22*^/+ flies were quantitatively similar, demonstrating that dTau is unlikely to be the substrate of Par-1 that mediates the axonal transport deficits elicited by elevated levels of Par-1. Finally, we confirmed that BRP transport was unaffected in *tau*^*MR22*^ transheterozygotes as well as dtau^KO^ flies (S8 Fig). Together, these data strongly support the idea that BRP accumulations observed within the axons for Par-1 overexpressing flies are independent of Tau.

## Discussion

Our data suggest that elevated levels of Par-1 have a strong effect on the transport of an important active zone scaffolding protein-BRP, leading to defects in synaptic transmission. These data may have implications for neurodevelopmental disorders like autism spectrum disorders and neurodegenerative diseases where MARK/Par-1 levels are elevated[6–9, 11, 12]. Finally, our data presents convincing evidence for the existence of distinct pathways for the transport of different active zone proteins.

### Implications for neurodevelopmental disorders

MARK/Par-1 is an essential gene that is required for cell polarity[35, 42, 43] and therefore, is essential for proper embryogenesis. MARK/Par-1 is also enriched in neurons and has been shown to be important in neuronal development[44, 45]. Elegant studies in *C*. *elegans* have shown that SYD2 and Liprin-α- two active zone proteins- are important in synapse assembly and maturation[23, 46, 47]. Interestingly, while SYD2/ Liprin-α can interact with (Rab3 interacting molecule) RIM/Unc10[48] respectively these interactions are dispensable for active zone maturation[23]. The maturation of active zones instead depends on the interaction with BRP homolog, ELKS both in *C*. *elegans* and mice[23, 49]. Since our data shows that axonal transport of BRP is deficient in Par-1 overexpressing flies, this may affect the development or maturation of active zone, which may in turn have functional consequences as suggested by our data that shows reduced synaptic transmission in flies overexpressing Par-1. Decrease in number of T-bars in Par-1 overexpression flies is not accompanied by a change in apposition of active zones and PSDs suggesting a developmental defect and such a defect could arise due the synaptic instability[50, 51]. Finally, MARK/Par-1 levels are increased in postmortem brains of children diagnosed with ASD[6]. Importantly, the increase in MARK is specific to pre-frontal cortex in ASD, a region of brain most affected in ASDs[6]. Our data would suggest that increase in MARK/Par-1 might lead to defects in active zone formation or maturation in these areas. These questions need to be addressed by future studies.

### Implications for neurodegenerative diseases

Hyperphosphorylation of Tau has been hypothesized to be the underlying cause of neurodegeneration[13]. MARK/Par-1 can phosphorylate Tau at serine 262[31], which has been shown to be hyperphosphorylated in postmortem Alzheimer’s disease (AD) patient brains[52] and it has been demonstrated that MARK/Par-1 can function as a “initiator kinase”[9] for the cascade that hyperphosphorylates Tau. *In vitro*, hyperphosphorylation of Tau can cause its detachment from microtubules leading to their destabilization[32]. Microtubules serve as “highways” on which the transport of synaptic cargo is dependent[30]. Thus, overexpression of Par-1 could lead to hyperphosphorylation of Tau and could cause axonal transport deficits. Our data suggest that axonal transport defects caused due to Par-1 overexpression are independent of endogenous *Drosophila* Tau (dTau). Although *Drosophila* Par-1 can phosphorylate Tau *in vitro[9]*, previous studies have shown that it can act independently of Tau *in vivo[35]*, suggesting additional substrates of Par-1 could possibly regulate specific transport of active zone protein, BRP. Furthermore, many studies suggest that axonal transport is likely to precede overt neurodegeneration[53–56]. It is tempting to speculate that one possibility is that transport of active zone proteins could be an initial event that leads to synaptic dysfunction, another symptom that precedes neuronal degeneration[57]. Thus, although the ultimate neuronal degeneration could be driven by hyperphosphorylation of Tau, other proteins may play a role in setting the stage for Tau pathology. Indeed, *in vivo* observation of axonal transport in a mouse model of neurodegeneration suggests that axonal transport can occur early in the neuronal pathology and is likely not driven by Tau[58].

### Implications for transport of active zone proteins

PTVs (Piccolo-Bassoon transport vesicles) have been shown to carry largely active zone components[59] in mammalian cell culture studies. However, some of the main components of PTVs for example, Basoon have no homologs in invertebrates[16]. Recent studies in flies and *C*. *elegans* have shed some light on the mechanisms of active zone transport. For example, mutants in imac (kinesin 3 homolog in flies) have severe reductions in BRP protein at the synapses[60]. However, these flies also have reduction in synaptic vesicles[60], suggesting that imac may transport both synaptic vesicles and active zone components. Supporting this argument, studies in *C*. *elegans* show that synaptic vesicles and active zone components are transported together[61]. However, a recent study in flies suggests that active zone components could be transported in distinct vesicles[62]. This study found that BRP and RIM-binding protein (RBP) can be co-transported[62]. Intriguingly, RBP and BRP transport could be uncoupled[62]. Indeed, our data supports such an idea and suggests the possibility that BRP could be transported via a distinct mechanism. Overexpression of Par-1 leads to specific accumulation of BRP while mitochondria; markers for synaptic vesicles and other active zone proteins do not accumulate. Our data also demonstrate that this process is not mediated Tau. One possible target of Par-1 as suggested earlier[35] is another microtubule binding protein Futsch[63]. Intriguingly, presynaptic reduction of Futsch leads to a reduction in active zone numbers and leads to defects in neurotransmission[64]. This possibly remains to be determined.

Par-1 phosphorylates a conserved KXGS motif and our initial analysis suggests that Futsch and its vertebrate homolog, MAP1B[63] contains one KXGS motif. Interestingly, Discs Large (Dlg), a homolog of PSD-95[18] is also phosphorylated by Par-1 kinase[15] and Dlg also contains only one KXGS motif that can be phosphorylated, suggesting that presence of single KXGS motif might be enough for Par-1 to phosphorylate a protein. Further analysis is required to test whether Futsch may be involved in the regulation of transport of BRP. Interestingly, at the synapse, Futsch is present closer to BRP than microtubules[64] thus making it a plausible target for mediating the transport of BRP.

Since the transport defects we observe are so specific one alternative is that Par-1 directly phosphorylates BRP. Similar to Futsch, BRP also has a KXGS motif that is present at its conserved N-terminus. Thus, Par-1 could phosphorylate BRP and directly affect its transport. Since the N-terminus of BRP is more conserved with the vertebrate ERC2 and C. *elegans* ELKS protein[16], it is likely that such a mechanism might also be conserved. These intriguing possibilities should be explored in future studies.

### What are the upstream regulators of Par-1?

Par-1 is activated by LKB1 by phosphorylating it on the threonine 408. Our data suggest that Threonine 408 is necessary for the manifestation of BRP transport phenotype. Overexpression of inactive Par-1 (Par-1T^408A^) does not lead to BRP accumulation within axons, suggesting that inactive Par-1 cannot induce the accumulation of BRP within the axons. However, overexpression of LKB1 in neurons is unable to induce accumulation of BRP within axons (S9 Fig) suggesting that while activation of Par-1 by LKB1 might be indeed important in increasing the toxicity of Tau[12], it may not be necessary to induce BRP accumulation in the axons. Furthermore, this raises the possibility of a novel upstream regulator of Par-1 kinase that might be important in regulating the transport of BRP within axons.

Thus, our current study demonstrates that distinct mechanisms exist to transport components of active zones like BRP and that availability of these components is likely regulated tightly by kinases such as Par-1 kinase.

## Materials and methods

### Fly stocks

Flies were reared in medium containing Nutri-Fly^TM^ Bloomington formulation (Genesee Scientific, San Diego, CA), Jazz mix (Fisher Scientific, Waltham, MA, USA), sugar and powdered yeast (Genesee Scientific) in an 8:5:1:1 ratio and made according to standard procedures. The following fly stocks were used in this study: UAS-Par-1[12], UAS-Par-1^T408A^ [22], BG380-Gal4[18], Df(3R)tauMR22[34, 35], UAS-GFP[65], and UAS-mito-GFP[66]. BG380-Gal4 was obtained from Aaron DiAntonio, Washington University Medical School (St. Louis, MO, USA). Df(3R)tauMR22 was a generous gift from Daniel St. Johnston, University of Cambridge (UK). UAS-Par-1 and UAS-PAR-1^T408A^ were obtained from Bingwei Lu, Stanford School of Medicine (Stanford, CA, USA).

### Immunohistochemistry

Larvae were dissected and stained as described previously[27, 67]. Following primary antibodies were used: anti-BRP (1:250)[16], anti-Tubulin (E7) (1:100) (obtained from the Developmental Studies Hybridoma Bank), anti-GFP (1:500)[41] (obtained from abcam), anti-DVGLUT (1:10,000)[19](gift from Aaron Diantonio, Washington University Medical School), anti-Liprin-α (1:500)[68](gift from Stephan Sigrist, Free University Berlin), anti-DAB [24](gift from Richard Ordway, Pennsylvania State University), and anti-dTau (1:1000)[34, 35](gift from Doris Kretzschmar, Oregon Health and Science University and Daniel St. Johnston, University of Cambridge). Dylight conjugated goat anti-HRP antibody (1:1,000), Goat Cy3-, and Alexa 488 conjugated secondary antibodies against mouse, rabbit, and chicken IgG (1:1000) were obtained from Jackson ImmunoResearch, West Grove, PA.

### Imaging and analysis

All axonal imaging was done between segments A2–A4. All the NMJ imaging was done at muscle 4, segment A2 –A4. Imaging and analysis of intensity of proteins within axons were done as described previously[26]. Staining intensities of various proteins within the axons and the NMJs were quantified by using MetaMorph software (Molecular Devices, Sunnyvale, CA, USA). For axons and NMJs, HRP was used to set the color threshold. Only the axonal compartment and NMJ region was used to measure the intensity of the red, green and blue (HRP) channels. The intensity of HRP did not vary significantly within the same experimental group. Active zones were counted manually by counting the puncta stained by an anti-BRP antibody. Statistical analysis and graphs were generated using GraphPad Prism (GraphPad Software, Inc.). Student T-tests and One-way ANOVA followed by Dunnett’s or Tukey’s multiple comparison tests were performed to compare each group with other samples.

### Electrophysiology

Intracellular electrophysiological recordings were performed on muscle 6, segment A2-A4 as previously described[69]. Quantal content was determined by dividing the mean EJP amplitude by the mean mEJP amplitude (EJP/ mEJP). The cells across all genotypes had similar mean input resistances and resting membrane potentials. Statistical analysis and graphs were generated using GraphPad Prism (GraphPad Software, Inc.). One-way ANOVA followed by Dunnett’s or Tukey’s multiple comparison tests were performed to compare each group with other samples.

### Western blots

Western blots were performed as described in[28] and run on 8% SDS-PAGE gels. Briefly, heads of flies were separated manually and 20 heads were used to extract lysates using 1x SDS buffer. 6 head equivalent lysate was loaded into each well and probed for dTau using anti-dTau antibody(1:10,000) [34, 35] (gift from Doris Kretzschmar, Oregon Health and Science University and Daniel St. Johnston, University of Cambridge). 30 head equivalent lysate against anti- BRP (1:100)[16] (Developmental Studies Hybridoma Bank) and anti-Tau (phospho S262) (1:1000) (abcam)[9] were performed according to Gorska-Andrzejak et al [70]. In all experiments Syx1A Antibody (8C3) (1:100)[71](Developmental Studies Hybridoma Bank) was used as a loading control. Image J was used to analyze the intensity of bands on the western blots and the “Gel analysis” function in the program was used to quantify the intensity of the bands. Ratios of the intensities of WT, Par-1^OE^, or Par-1^T408A^ bands to that of Syntaxin bands were measured and used for calculating the statistical differences between the genotypes. Statistical analysis was generated using GraphPad Prism (GraphPad Software, Inc.). Student T-tests and one-way ANOVA followed by Dunnett’s or Tukey’s multiple comparison tests were performed to compare each group with other samples.

### Electron microscopy

Samples for ultrastructural analysis were performed as previously described[28]. The larval head and tail were pinned and a dorsal slit was made lengthwise, thus filleting the larvae–in Tannic acid. The larvae were then post-fixed in 1% osmium tetroxide for 1 hr at 4°C. The larvae were dehydrated through 60, (1x, 7 min) 70, 80, 95 and 100% EtOH (2x, 10 min each step), transferred into propylene oxide (2x, 10 min), then into a 1:1 mixture of propylene oxide and Eponate, and left o/n, capped and at room temp. The larvae were then placed into fresh Eponate and into a mould, oriented and allowed to polymerize at 70°C. Thin sections were made and placed on superfrost/plus micro slide and stained with Toluidine Blue “O”. Type 1b boutons from NMJ6/7 in segment A2-A4 from WT and Par-1^OE^ larval neuromuscular junctions were identified from the thin sections. Sections were cut at 50 nm with a diamond knife, picked up on formvar coated, copper slot grids, and stained with 2% aqueous uranyl acetate for 15 min followed by lead citrate stain for 1 min. Samples were observed and photographed in a JEM-1400 (JOEL, Japan) transmission electron microscope. Active zones and T-bars were quantified manually. Statistical analysis and graphs were generated using GraphPad Prism (GraphPad Software, Inc.). One-way ANOVA followed by Dunnett’s or Tukey’s multiple comparison tests were performed to compare each group with other samples.

## Supporting information

S1 Text Supplemental Materials and Methods. (DOCX)

S1 Fig Most tested presynaptic drivers show an increased accumulation of BRP in axons. **A**) Representative confocal image stacks showing axons from WT and Par-1^OE^ third instar larvae stained with antibodies against BRP (red) and HRP (Blue) using different presynaptic Gal-4 drivers (indicated on figure). **B**) Quantification of BRP intensity from axons from genotypes in A. n = 10, **** = p<0.0001. Scale Bar = 10μm. Error bars represent S.E.M. **C**) Representative confocal image stacks showing axons from WT and Par-1^OE^ third instar larvae stained with antibodies against BRP (red) and HRP (Blue) using postsynaptic drive G7-Gal-4. N = 10, p = 0.47. Scale Bar = 10μm.
(TIFF)

S2 Fig Overexpression of Par-1 does not lead to changes in synaptic span or apposition but leads to a significant decrease in bouton size. **A**) Representative confocal image stacks showing NMJs from WT, Par-1^OE^ and Par-1^T408A^ third instar larvae stained with antibodies against BRP (red), DGluRIII (Green) and HRP (Blue). Synaptic apposition as marked by the apposition of BRP and DGluRIII (Inset) was unchanged. Scale Bar = 5μm. **B**) Quantification of Synaptic Span. N = 10, p = 0.39. **C**) Quantification of bouton area. N = 10, **** = p<0.0001. Error bars represent S.E.M.
(TIFF)

S3 Fig Levels of other synaptic proteins are unaffected at synapses in flies overexpressing Par-1. **A**) Representative confocal image stacks showing NMJ synapses from WT and **B**) Par-1^OE^ third instar larvae stained against Liprin-α, DAB (Green) and HRP (Blue). Scale bar = 10μm.). **C**) Quantification of Liprin-α (Green) intensity at synapses. N = 12, p = 0.49. Error bars represent S.E.M. **D**) Quantification of DAB (Green) intensity at synapses. N = 12, p = 0.09. Error bars represent S.E.M. **E**) Quantification of Mitochondria area within axons (see Fig 2) of WT and Par-1^OE^ larvae. N = 10, p = 0.7893. Error bars represent S.E.M. **F**) Quantification showing the ratio of Liprin-α intensity at axons and synapses. N = 12, p = 0.1425. Error bars represent S.E.M. **G**) Quantification showing the ratio of DAB intensity at axons and synapses. N = 12, p = 0.1354. Error bars represent S.E.M. H) Quantification of showing the ration of BRP intensity at axons and synapses WT and Par-1^OE^ larvae. N = 10, **** = p<0.0001.
(TIFF)

S4 Fig When Par-1 is overexpressed it prominently localizes within axons. Representative images from WT and Par-1^OE^ and Par-1^T408A^ flies showing localization of overexpressed Par-1 (Red), endogenous tau (Green), and HRP (Blue) in axons.
(TIF)

S5 Fig Validation of dTau antibody within axons. **A**) Representative images from WT and Par-1^OE^ flies showing localization of overexpressed Tau^GFP^ (using anti-GFP antibody, Red) and dTau antibody (Green) in axons. Scale Bar = 10μm. **B**) Representative images from WT and d*tau*^KO^ flies showing localization of dTau (Green) in axons and HRP (Blue). Scale Bar = 10μm.
(TIFF)

S6 Fig Overexpression of dTau does not affect the transport of BRP. **A**) Representative confocal image stacks showing axons from WT and tau^GFP^ stained with antibodies against BRP (Red) and HRP (Blue). **B**) Quantification of BRP intensity in axons of identical genotypes as in A. N = 10, p = 0.30. Error bars represent S.E.M. C) Quantification of BRP puncta size within axons of identical genotypes seen in A. N = 10, p = 0.52. Error bars represent S.E.M.
(TIFF)

S7 Fig t*au^MR22^* heterozygotes have a significant reduction of dTau in axons. **A**) Representative images from WT and *tau*^*MR22*^/+ flies showing axons stained against dTau (Green) and HRP (Blue). **B**) Quantification of dTau intensity in WT and *tau*^*MR22*^/+ axons. N = 8, **** = p<0.0001. Scale bar = 10μm. Error bars represent S.E.M.
(TIFF)

S8 Fig BRP transport was unaffected in *tau^MR22^* transheterozygotes as well as dtau^KO^ flies. **A**) Representative images from WT and *tau*^*MR22*^/+ flies showing axons stained against BRP (Red) and HRP (Blue). **B**) Quantification of BRP intensity in axons. N = 8, p = 0.4309. Scale bar = 10μm. **C**) Representative images from WT and dtau^KO^ flies showing axons stained against BRP (Red) and HRP (Blue). **D**) Quantification of BRP intensity in axons. N = 8, p = 0.24. Scale bar = 10μm. Error bars represent S.E.M.
(TIFF)

S9 Fig Overexpression of LKB1 in neurons is unable to induce accumulation of BRP within axons. **A**) Representative images from WT and presynaptic overexpression of LKB1 (LKB1^OE^) flies driven using BG380-Gal4 showing axons stained against BRP (Red) and HRP (Blue). **B**) Quantification of BRP intensity in axons. N = 8, p = 0.58. Scale bar = 10μm. Error bars represent S.E.M.
(TIFF)

## References

[1] Sudhof TC. The presynaptic active zone. Neuron. 2012;75(1):11–25. PubMed Central PMCID: PMCPMC3743085. 10.1016/j.neuron.2012.06.012 22794257 PMC3743085

[2] Zhai RG, Bellen HJ. The architecture of the active zone in the presynaptic nerve terminal. Physiology (Bethesda). 2004;19:262–70. 15381754 10.1152/physiol.00014.2004

[3] Zoghbi HY, Bear MF. Synaptic dysfunction in neurodevelopmental disorders associated with autism and intellectual disabilities. Cold Spring Harb Perspect Biol. 2012;4(3). PubMed Central PMCID: PMCPMC3282414. 10.1101/cshperspect.a009886 PMC3282414 22258914

[4] Bourgeron T. A synaptic trek to autism. Curr Opin Neurobiol. 2009;19(2):231–4. 10.1016/j.conb.2009.06.003 19545994

[5] Spires-Jones TL, Hyman BT. The intersection of amyloid beta and tau at synapses in Alzheimer's disease. Neuron. 2014;82(4):756–71. Epub 2014/05/24. PubMed Central PMCID: PMC4135182. 10.1016/j.neuron.2014.05.004 24853936 PMC4135182

[6] Maussion G, Carayol J, Lepagnol-Bestel AM, Tores F, Loe-Mie Y, Milbreta U, et al Convergent evidence identifying MAP/microtubule affinity-regulating kinase 1 (MARK1) as a susceptibility gene for autism. Hum Mol Genet. 2008;17(16):2541–51. 10.1093/hmg/ddn154 18492799

[7] Hu VW, Sarachana T, Kim KS, Nguyen A, Kulkarni S, Steinberg ME, et al Gene expression profiling differentiates autism case-controls and phenotypic variants of autism spectrum disorders: evidence for circadian rhythm dysfunction in severe autism. Autism Res. 2009;2(2):78–97. PubMed Central PMCID: PMCPMC2737477. 10.1002/aur.73 19418574 PMC2737477

[8] Carayol J, Schellenberg GD, Dombroski B, Genin E, Rousseau F, Dawson G. Autism risk assessment in siblings of affected children using sex-specific genetic scores. Mol Autism. 2011;2(1):17 PubMed Central PMCID: PMCPMC3214848. 10.1186/2040-2392-2-17 22017886 PMC3214848

[9] Nishimura I, Yang Y, Lu B. PAR-1 kinase plays an initiator role in a temporally ordered phosphorylation process that confers tau toxicity in Drosophila. Cell. 2004;116(5):671–82. Epub 2004/03/10. 15006350 10.1016/s0092-8674(04)00170-9

[10] Yu W, Polepalli J, Wagh D, Rajadas J, Malenka R, Lu B. A critical role for the PAR-1/MARK-tau axis in mediating the toxic effects of Abeta on synapses and dendritic spines. Hum Mol Genet. 2012;21(6):1384–90. Epub 2011/12/14. PubMed Central PMCID: PMC3284124. 10.1093/hmg/ddr576 22156579 PMC3284124

[11] Chin JY, Knowles RB, Schneider A, Drewes G, Mandelkow EM, Hyman BT. Microtubule-affinity regulating kinase (MARK) is tightly associated with neurofibrillary tangles in Alzheimer brain: a fluorescence resonance energy transfer study. Journal of neuropathology and experimental neurology. 2000;59(11):966–71. Epub 2000/11/23. 11089574 10.1093/jnen/59.11.966

[12] Lee S, Wang JW, Yu W, Lu B. Phospho-dependent ubiquitination and degradation of PAR-1 regulates synaptic morphology and tau-mediated Abeta toxicity in Drosophila. Nature communications. 2012;3:1312 Epub 2012/12/29. PubMed Central PMCID: PMC4307937. 10.1038/ncomms2278 23271647 PMC4307937

[13] Braak H, Del Tredici K. The preclinical phase of the pathological process underlying sporadic Alzheimer's disease. Brain. 2015;138(Pt 10):2814–33. 10.1093/brain/awv236 26283673

[14] Lund H, Gustafsson E, Svensson A, Nilsson M, Berg M, Sunnemark D, et al MARK4 and MARK3 associate with early tau phosphorylation in Alzheimer's disease granulovacuolar degeneration bodies. Acta Neuropathol Commun. 2014;2:22 PubMed Central PMCID: PMCPMC4046661. 10.1186/2051-5960-2-22 24533944 PMC4046661

[15] Zhang Y, Guo H, Kwan H, Wang JW, Kosek J, Lu B. PAR-1 kinase phosphorylates Dlg and regulates its postsynaptic targeting at the Drosophila neuromuscular junction. Neuron. 2007;53(2):201–15. Epub 2007/01/17. PubMed Central PMCID: PMC1855201. 10.1016/j.neuron.2006.12.016 17224403 PMC1855201

[16] Wagh DA, Rasse TM, Asan E, Hofbauer A, Schwenkert I, Durrbeck H, et al Bruchpilot, a protein with homology to ELKS/CAST, is required for structural integrity and function of synaptic active zones in Drosophila. Neuron. 2006;49(6):833–44. Epub 2006/03/18. 10.1016/j.neuron.2006.02.008 16543132

[17] Brand AH, Perrimon N. Targeted gene expression as a means of altering cell fates and generating dominant phenotypes. Development. 1993;118(2):401–15. Epub 1993/06/01. 8223268 10.1242/dev.118.2.401

[18] Budnik V, Koh YH, Guan B, Hartmann B, Hough C, Woods D, et al Regulation of synapse structure and function by the Drosophila tumor suppressor gene dlg. Neuron. 1996;17(4):627–40. PubMed Central PMCID: PMCPMC4661176. 8893021 10.1016/s0896-6273(00)80196-8 PMC4661176

[19] Daniels RW, Collins CA, Gelfand MV, Dant J, Brooks ES, Krantz DE, et al Increased expression of the Drosophila vesicular glutamate transporter leads to excess glutamate release and a compensatory decrease in quantal content. The Journal of neuroscience: the official journal of the Society for Neuroscience. 2004;24(46):10466–74. Epub 2004/11/19. 15548661 10.1523/JNEUROSCI.3001-04.2004 PMC6730318

[20] Jan LY, Jan YN. Antibodies to horseradish peroxidase as specific neuronal markers in Drosophila and in grasshopper embryos. Proc Natl Acad Sci U S A. 1982;79(8):2700–4. PubMed Central PMCID: PMCPMC346269. 6806816 10.1073/pnas.79.8.2700 PMC346269

[21] Zhang YQ, Bailey AM, Matthies HJ, Renden RB, Smith MA, Speese SD, et al Drosophila fragile X-related gene regulates the MAP1B homolog Futsch to control synaptic structure and function. Cell. 2001;107(5):591–603. 11733059 10.1016/s0092-8674(01)00589-x

[22] Wang JW, Imai Y, Lu B. Activation of PAR-1 kinase and stimulation of tau phosphorylation by diverse signals require the tumor suppressor protein LKB1. The Journal of neuroscience: the official journal of the Society for Neuroscience. 2007;27(3):574–81. Epub 2007/01/20. 17234589 10.1523/JNEUROSCI.5094-06.2007 PMC6672797

[23] Dai Y, Taru H, Deken SL, Grill B, Ackley B, Nonet ML, et al SYD-2 Liprin-alpha organizes presynaptic active zone formation through ELKS. Nat Neurosci. 2006;9(12):1479–87. 10.1038/nn1808 17115037

[24] Kawasaki F, Iyer J, Posey LL, Sun CE, Mammen SE, Yan H, et al The DISABLED protein functions in CLATHRIN-mediated synaptic vesicle endocytosis and exoendocytic coupling at the active zone. Proc Natl Acad Sci U S A. 2011;108(25):E222–9. PubMed Central PMCID: PMCPMC3121831. 10.1073/pnas.1102231108 21606364 PMC3121831

[25] Glater EE, Megeath LJ, Stowers RS, Schwarz TL. Axonal transport of mitochondria requires milton to recruit kinesin heavy chain and is light chain independent. J Cell Biol. 2006;173(4):545–57. PubMed Central PMCID: PMCPMC2063864. 10.1083/jcb.200601067 16717129 PMC2063864

[26] Wairkar YP, Trivedi D, Natarajan R, Barnes K, Dolores L, Cho P. CK2alpha regulates the transcription of BRP in Drosophila. Developmental biology. 2013;384(1):53–64. Epub 2013/10/02. 10.1016/j.ydbio.2013.09.025 24080510

[27] Marrus SB, Portman SL, Allen MJ, Moffat KG, DiAntonio A. Differential localization of glutamate receptor subunits at the Drosophila neuromuscular junction. The Journal of neuroscience: the official journal of the Society for Neuroscience. 2004;24(6):1406–15. Epub 2004/02/13. 14960613 10.1523/JNEUROSCI.1575-03.2004 PMC6730334

[28] Wairkar YP, Toda H, Mochizuki H, Furukubo-Tokunaga K, Tomoda T, Diantonio A. Unc-51 controls active zone density and protein composition by downregulating ERK signaling. The Journal of neuroscience: the official journal of the Society for Neuroscience. 2009;29(2):517–28. PubMed Central PMCID: PMCPMC2741695. 19144852 10.1523/JNEUROSCI.3848-08.2009 PMC2741695

[29] Petersen SA, Fetter RD, Noordermeer JN, Goodman CS, DiAntonio A. Genetic analysis of glutamate receptors in Drosophila reveals a retrograde signal regulating presynaptic transmitter release. Neuron. 1997;19(6):1237–48. Epub 1998/01/14. 9427247 10.1016/s0896-6273(00)80415-8

[30] Goldstein AY, Wang X, Schwarz TL. Axonal transport and the delivery of pre-synaptic components. Curr Opin Neurobiol. 2008;18(5):495–503. PubMed Central PMCID: PMCPMC2653082. 10.1016/j.conb.2008.10.003 18950710 PMC2653082

[31] Drewes G, Trinczek B, Illenberger S, Biernat J, Schmitt-Ulms G, Meyer HE, et al Microtubule-associated protein/microtubule affinity-regulating kinase (p110mark). A novel protein kinase that regulates tau-microtubule interactions and dynamic instability by phosphorylation at the Alzheimer-specific site serine 262. J Biol Chem. 1995;270(13):7679–88. 7706316 10.1074/jbc.270.13.7679

[32] Drewes G, Ebneth A, Preuss U, Mandelkow EM, Mandelkow E. MARK, a novel family of protein kinases that phosphorylate microtubule-associated proteins and trigger microtubule disruption. Cell. 1997;89(2):297–308. Epub 1997/04/18. 9108484 10.1016/s0092-8674(00)80208-1

[33] Mandelkow EM, Thies E, Trinczek B, Biernat J, Mandelkow E. MARK/PAR1 kinase is a regulator of microtubule-dependent transport in axons. J Cell Biol. 2004;167(1):99–110. Epub 2004/10/07. PubMed Central PMCID: PMC2172520. 10.1083/jcb.200401085 15466480 PMC2172520

[34] Bolkan BJ, Kretzschmar D. Loss of Tau results in defects in photoreceptor development and progressive neuronal degeneration in Drosophila. Dev Neurobiol. 2014;74(12):1210–25. PubMed Central PMCID: PMCPMC4212004. 10.1002/dneu.22199 24909306 PMC4212004

[35] Doerflinger H, Benton R, Shulman JM, St Johnston D. The role of PAR-1 in regulating the polarised microtubule cytoskeleton in the Drosophila follicular epithelium. Development. 2003;130(17):3965–75. Epub 2003/07/23. 12874119 10.1242/dev.00616

[36] Iijima-Ando K, Sekiya M, Maruko-Otake A, Ohtake Y, Suzuki E, Lu B, et al Loss of axonal mitochondria promotes tau-mediated neurodegeneration and Alzheimer's disease-related tau phosphorylation via PAR-1. PLoS Genet. 2012;8(8):e1002918 PubMed Central PMCID: PMCPMC3431335. 10.1371/journal.pgen.1002918 22952452 PMC3431335

[37] Wolf N, Regan CL, Fuller MT. Temporal and spatial pattern of differences in microtubule behaviour during Drosophila embryogenesis revealed by distribution of a tubulin isoform. Development. 1988;102(2):311–24. 3138100 10.1242/dev.102.2.311

[38] Burnouf S, Gronke S, Augustin H, Dols J, Gorsky MK, Werner J, et al Deletion of endogenous Tau proteins is not detrimental in Drosophila. Sci Rep. 2016;6:23102 PubMed Central PMCID: PMCPMC4792132. 10.1038/srep23102 26976084 PMC4792132

[39] Andorfer C, Kress Y, Espinoza M, de Silva R, Tucker KL, Barde YA, et al Hyperphosphorylation and aggregation of tau in mice expressing normal human tau isoforms. J Neurochem. 2003;86(3):582–90. 12859672 10.1046/j.1471-4159.2003.01879.x

[40] Adams SJ, Crook RJ, Deture M, Randle SJ, Innes AE, Yu XZ, et al Overexpression of wild-type murine tau results in progressive tauopathy and neurodegeneration. Am J Pathol. 2009;175(4):1598–609. PubMed Central PMCID: PMCPMC2751556. 10.2353/ajpath.2009.090462 19717642 PMC2751556

[41] Gallio M, Ofstad TA, Macpherson LJ, Wang JW, Zuker CS. The coding of temperature in the Drosophila brain. Cell. 2011;144(4):614–24. PubMed Central PMCID: PMCPMC3336488. 10.1016/j.cell.2011.01.028 21335241 PMC3336488

[42] Doerflinger H, Benton R, Torres IL, Zwart MF, St Johnston D. Drosophila anterior-posterior polarity requires actin-dependent PAR-1 recruitment to the oocyte posterior. Curr Biol. 2006;16(11):1090–5. 10.1016/j.cub.2006.04.001 16753562

[43] Huynh JR, Shulman JM, Benton R, St Johnston D. PAR-1 is required for the maintenance of oocyte fate in Drosophila. Development. 2001;128(7):1201–9. 11245586 10.1242/dev.128.7.1201

[44] Tabler JM, Yamanaka H, Green JB. PAR-1 promotes primary neurogenesis and asymmetric cell divisions via control of spindle orientation. Development. 2010;137(15):2501–5. 10.1242/dev.049833 20573701

[45] Ossipova O, Ezan J, Sokol SY. PAR-1 phosphorylates Mind bomb to promote vertebrate neurogenesis. Dev Cell. 2009;17(2):222–33. PubMed Central PMCID: PMCPMC2849776. 10.1016/j.devcel.2009.06.010 19686683 PMC2849776

[46] Zhen M, Jin Y. The liprin protein SYD-2 regulates the differentiation of presynaptic termini in C. elegans. Nature. 1999;401(6751):371–5. 10.1038/43886 10517634

[47] Taru H, Jin Y. The Liprin homology domain is essential for the homomeric interaction of SYD-2/Liprin-alpha protein in presynaptic assembly. The Journal of neuroscience: the official journal of the Society for Neuroscience. 2011;31(45):16261–8. PubMed Central PMCID: PMCPMC3500560. 22072677 10.1523/JNEUROSCI.0002-11.2011 PMC3500560

[48] Stigloher C, Zhan H, Zhen M, Richmond J, Bessereau JL. The presynaptic dense projection of the Caenorhabditis elegans cholinergic neuromuscular junction localizes synaptic vesicles at the active zone through SYD-2/liprin and UNC-10/RIM-dependent interactions. The Journal of neuroscience: the official journal of the Society for Neuroscience. 2011;31(12):4388–96. PubMed Central PMCID: PMCPMC3077722. 21430140 10.1523/JNEUROSCI.6164-10.2011 PMC3077722

[49] Chia PH, Li P, Shen K. Cell biology in neuroscience: cellular and molecular mechanisms underlying presynapse formation. J Cell Biol. 2013;203(1):11–22. PubMed Central PMCID: PMCPMC3798257. 10.1083/jcb.201307020 24127213 PMC3798257

[50] Eaton BA, Davis GW. Synapse disassembly. Genes & development. 2003;17(17):2075–82. Epub 2003/09/04. 12952887 10.1101/gad.1113703

[51] Eaton BA, Fetter RD, Davis GW. Dynactin is necessary for synapse stabilization. Neuron. 2002;34(5):729–41. Epub 2002/06/14. 12062020 10.1016/s0896-6273(02)00721-3

[52] Gu GJ, Wu D, Lund H, Sunnemark D, Kvist AJ, Milner R, et al Elevated MARK2-dependent phosphorylation of Tau in Alzheimer's disease. J Alzheimers Dis. 2013;33(3):699–713. 10.3233/JAD-2012-121357 23001711

[53] Bilsland LG, Sahai E, Kelly G, Golding M, Greensmith L, Schiavo G. Deficits in axonal transport precede ALS symptoms in vivo. Proc Natl Acad Sci U S A. 2010;107(47):20523–8. PubMed Central PMCID: PMCPMC2996651. 10.1073/pnas.1006869107 21059924 PMC2996651

[54] Tang Y, Scott DA, Das U, Edland SD, Radomski K, Koo EH, et al Early and selective impairments in axonal transport kinetics of synaptic cargoes induced by soluble amyloid beta-protein oligomers. Traffic. 2012;13(5):681–93. PubMed Central PMCID: PMCPMC3593673. 10.1111/j.1600-0854.2012.01340.x 22309053 PMC3593673

[55] Milde S, Adalbert R, Elaman MH, Coleman MP. Axonal transport declines with age in two distinct phases separated by a period of relative stability. Neurobiol Aging. 2015;36(2):971–81. PubMed Central PMCID: PMCPMC4321880. 10.1016/j.neurobiolaging.2014.09.018 25443288 PMC4321880

[56] Millecamps S, Julien JP. Axonal transport deficits and neurodegenerative diseases. Nature reviews Neuroscience. 2013;14(3):161–76. Epub 2013/01/31. 10.1038/nrn3380 23361386

[57] Yoshiyama Y, Higuchi M, Zhang B, Huang SM, Iwata N, Saido TC, et al Synapse loss and microglial activation precede tangles in a P301S tauopathy mouse model. Neuron. 2007;53(3):337–51. 10.1016/j.neuron.2007.01.010 17270732

[58] Majid T, Ali YO, Venkitaramani DV, Jang MK, Lu HC, Pautler RG. In vivo axonal transport deficits in a mouse model of fronto-temporal dementia. Neuroimage Clin. 2014;4:711–7. PubMed Central PMCID: PMCPMC4053640. 10.1016/j.nicl.2014.02.005 24936422 PMC4053640

[59] Zhai RG, Vardinon-Friedman H, Cases-Langhoff C, Becker B, Gundelfinger ED, Ziv NE, et al Assembling the presynaptic active zone: a characterization of an active one precursor vesicle. Neuron. 2001;29(1):131–43. Epub 2001/02/22. 11182086 10.1016/s0896-6273(01)00185-4

[60] Pack-Chung E, Kurshan PT, Dickman DK, Schwarz TL. A Drosophila kinesin required for synaptic bouton formation and synaptic vesicle transport. Nat Neurosci. 2007;10(8):980–9. 10.1038/nn1936 17643120

[61] Klassen MP, Wu YE, Maeder CI, Nakae I, Cueva JG, Lehrman EK, et al An Arf-like small G protein, ARL-8, promotes the axonal transport of presynaptic cargoes by suppressing vesicle aggregation. Neuron. 2010;66(5):710–23. PubMed Central PMCID: PMCPMC3168544. 10.1016/j.neuron.2010.04.033 20547129 PMC3168544

[62] Siebert M, Bohme MA, Driller JH, Babikir H, Mampell MM, Rey U, et al A high affinity RIM-binding protein/Aplip1 interaction prevents the formation of ectopic axonal active zones. eLife. 2015;4. Epub 2015/08/15. PubMed Central PMCID: PMC4536467. 10.7554/eLife.06935 PMC4536467 26274777

[63] Hummel T, Krukkert K, Roos J, Davis G, Klambt C. Drosophila Futsch/22C10 is a MAP1B-like protein required for dendritic and axonal development. Neuron. 2000;26(2):357–70. 10839355 10.1016/s0896-6273(00)81169-1

[64] Lepicard S, Franco B, de Bock F, Parmentier ML. A presynaptic role of microtubule-associated protein 1/Futsch in Drosophila: regulation of active zone number and neurotransmitter release. The Journal of neuroscience: the official journal of the Society for Neuroscience. 2014;34(20):6759–71. 24828631 10.1523/JNEUROSCI.4282-13.2014 PMC6608111

[65] Yeh E, Gustafson K, Boulianne GL. Green fluorescent protein as a vital marker and reporter of gene expression in Drosophila. Proc Natl Acad Sci U S A. 1995;92(15):7036–40. PubMed Central PMCID: PMCPMC41466. 7624365 10.1073/pnas.92.15.7036 PMC41466

[66] Pilling AD, Horiuchi D, Lively CM, Saxton WM. Kinesin-1 and Dynein are the primary motors for fast transport of mitochondria in Drosophila motor axons. Mol Biol Cell. 2006;17(4):2057–68. PubMed Central PMCID: PMCPMC1415296. 10.1091/mbc.E05-06-0526 16467387 PMC1415296

[67] Wairkar YP, Fradkin LG, Noordermeer JN, DiAntonio A. Synaptic defects in a Drosophila model of congenital muscular dystrophy. The Journal of neuroscience: the official journal of the Society for Neuroscience. 2008;28(14):3781–9. 18385336 10.1523/JNEUROSCI.0478-08.2008 PMC6671091

[68] Fouquet W, Owald D, Wichmann C, Mertel S, Depner H, Dyba M, et al Maturation of active zone assembly by Drosophila Bruchpilot. J Cell Biol. 2009;186(1):129–45. PubMed Central PMCID: PMCPMC2712991. 10.1083/jcb.200812150 19596851 PMC2712991

[69] Natarajan R, Trivedi-Vyas D, Wairkar YP. Tuberous sclerosis complex regulates Drosophila neuromuscular junction growth via the TORC2/Akt pathway. Hum Mol Genet. 2013;22(10):2010–23. Epub 2013/02/09. 10.1093/hmg/ddt053 23393158

[70] Gorska-Andrzejak J, Salvaterra PM, Meinertzhagen IA, Krzeptowski W, Gorlich A, Pyza E. Cyclical expression of Na+/K+-ATPase in the visual system of Drosophila melanogaster. J Insect Physiol. 2009;55(5):459–68. PubMed Central PMCID: PMCPMC2721802. 10.1016/j.jinsphys.2009.02.003 19428365 PMC2721802

[71] Burgess RW, Deitcher DL, Schwarz TL. The synaptic protein syntaxin1 is required for cellularization of Drosophila embryos. J Cell Biol. 1997;138(4):861–75. PubMed Central PMCID: PMCPMC2138053. 9265652 10.1083/jcb.138.4.861 PMC2138053

