# Correction: Active zone proteins are transported via distinct mechanisms regulated by Par-1 kinase

**DOI:** 10.1371/journal.pgen.1006822

**Published:** 2017-05-31

**Authors:** Kara R. Barber, Julia Tanquary, Keegan Bush, Amanda Shaw, Michael Woodson, Karthikeyan Tangavelou, Michael Sherman, Yogesh P. Wairkar

Dr. Karthikeyan Tangavelou should be included in the author byline. He should be listed as the sixth author, and his affiliation is 1: George and Cynthia Mitchell Center for Neurodegenerative Diseases, Department of Neurology, University of Texas Medical Branch, Galveston, TX, United States of America. The contributions of this author are as follows: Methodology, Investigation.

The correct citation is: Barber KR, Tanquary J, Bush K, Shaw A, Woodson M, Tangavelou K, et al. (2017) Active zone proteins are transported via distinct mechanisms regulated by Par-1 kinase. PLoS Genet 13(2): e1006621. doi:10.1371/journal.pgen.1006621
