# Supplementary material for: Active zone proteins are transported via distinct mechanisms regulated by Par-1 kinase
Source: PLoS Genet. 2017 Feb 21;13(2):e1006621. doi: 10.1371/journal.pgen.1006621 (PMC5340405; doi:10.1371/journal.pgen.1006621)
Supplement: S1 Text — (DOCX) [file pgen.1006621.s001.docx]

**Supplemental Materials and Methods**

**Fly Stocks**

Flies were reared as previously described in the main materials and methods section. Additional fly stocks that were used are as follows: elav-Gal4(72), nSyb-Gal4(73), G7-Gal4(21), UAS-tau-GFP(35), UAS-tau^KO^ (38), and UAS-LKB1(12). UAS-LKB1 was obtained from Bingwei Lu, Stanford School of Medicine (Stanford, CA, USA).

**Immunohistochemistry**

Larval dissection and staining were done as previously described. Additional antibodies used were as follows: anti-DGluRIII (1:1000)(69), and anti-Par-1 (1:10,000)(15)(gift from Bingwei Lu, Stanford School of Medicine).

**Imaging and analysis**

Axonal imaging and analysis were done as previously described. All axonal imaging was done between segments A2–A4. Bouton area, Mitochondrial area, and BRP puncta size was quantified manually using ImageJ (NIH). Synaptic span was quantified using the “Simple Neurite Tracer” plugin in Image J software. Statistical analysis and graphs were generated using GraphPad Prism (GraphPad Software, Inc.). Student T-tests and One-way ANOVA followed by Dunnett’s or Tukey’s multiple comparison tests were performed to compare each group with other samples.
