# Supplementary figures and images for: Active zone proteins are transported via distinct mechanisms regulated by Par-1 kinase
Source: PLoS Genet. 2017 Feb 21;13(2):e1006621. doi: 10.1371/journal.pgen.1006621 (PMC5340405; doi:10.1371/journal.pgen.1006621)

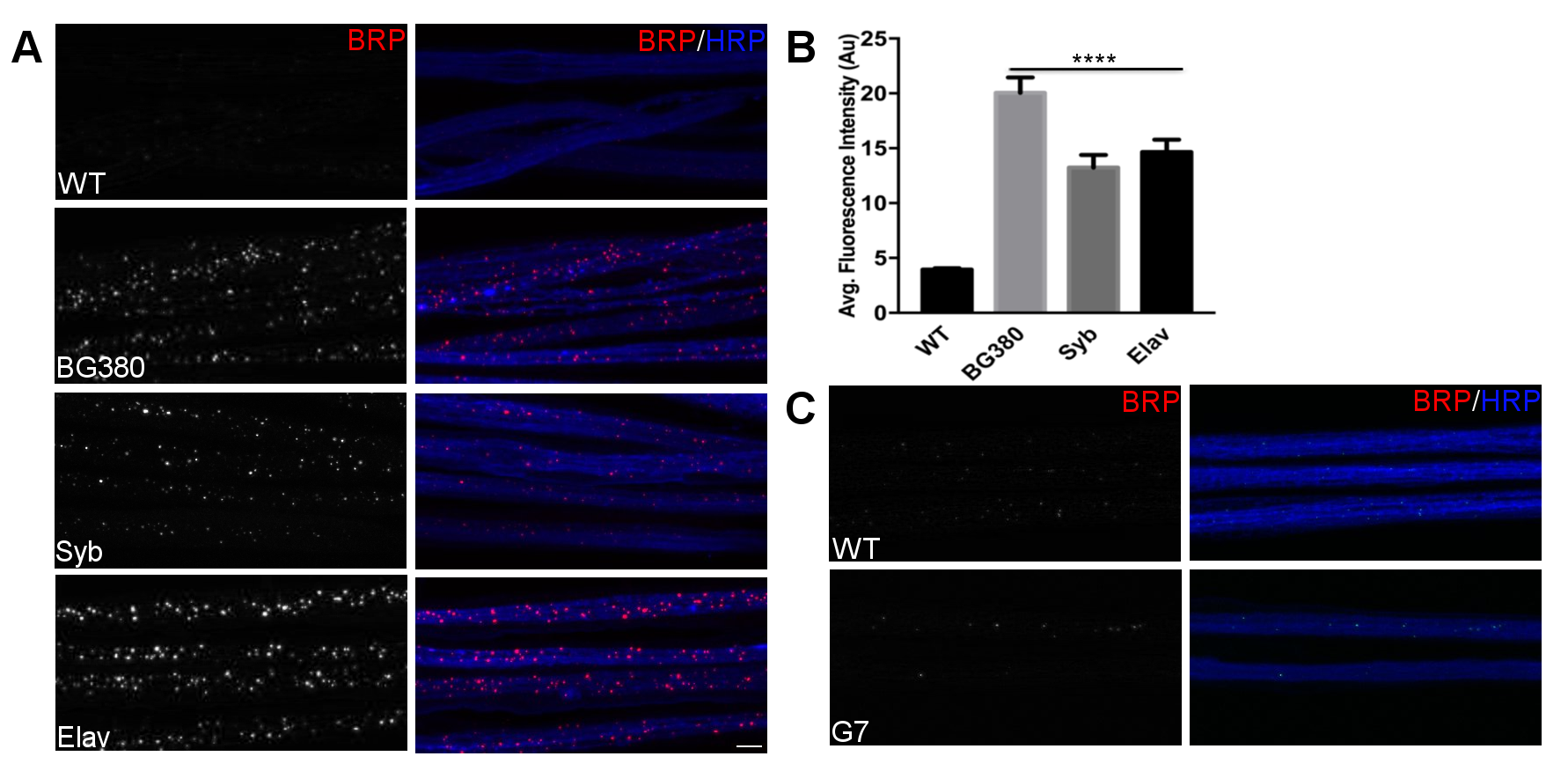

Supplement: S1 Fig — A) Representative confocal image stacks showing axons from WT and Par-1OE third instar larvae stained with antibodies against BRP (red) and HRP (Blue) using different presynaptic Gal-4 drivers (indicated on figure). B) Quantification of BRP intensity from axons from genotypes in A. n = 10, **** = p<0.0001. Scale Bar = 10μm. Error bars represent S.E.M. C) Representative confocal image stacks showing axons from WT and Par-1OE third instar larvae stained with antibodies against BRP (red) and HRP (Blue) using postsynaptic drive G7-Gal-4. N = 10, p = 0.47. Scale Bar = 10μm. (TIFF) [file pgen.1006621.s002.tiff]

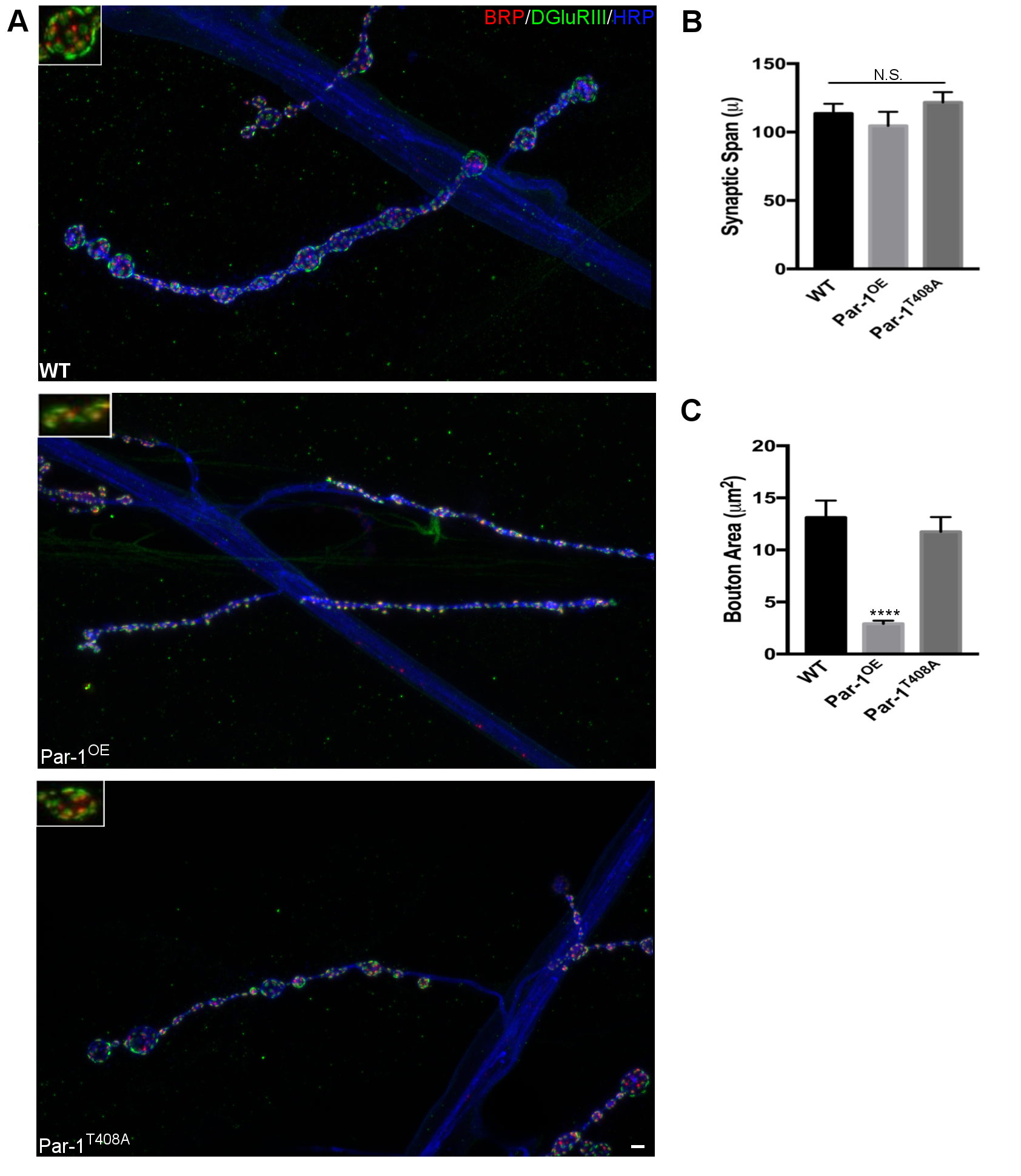

Supplement: S2 Fig — A) Representative confocal image stacks showing NMJs from WT, Par-1OE and Par-1T408A third instar larvae stained with antibodies against BRP (red), DGluRIII (Green) and HRP (Blue). Synaptic apposition as marked by the apposition of BRP and DGluRIII (Inset) was unchanged. Scale Bar = 5μm. B) Quantification of Synaptic Span. N = 10, p = 0.39. C) Quantification of bouton area. N = 10, **** = p<0.0001. Error bars represent S.E.M. (TIFF) [file pgen.1006621.s003.tiff]

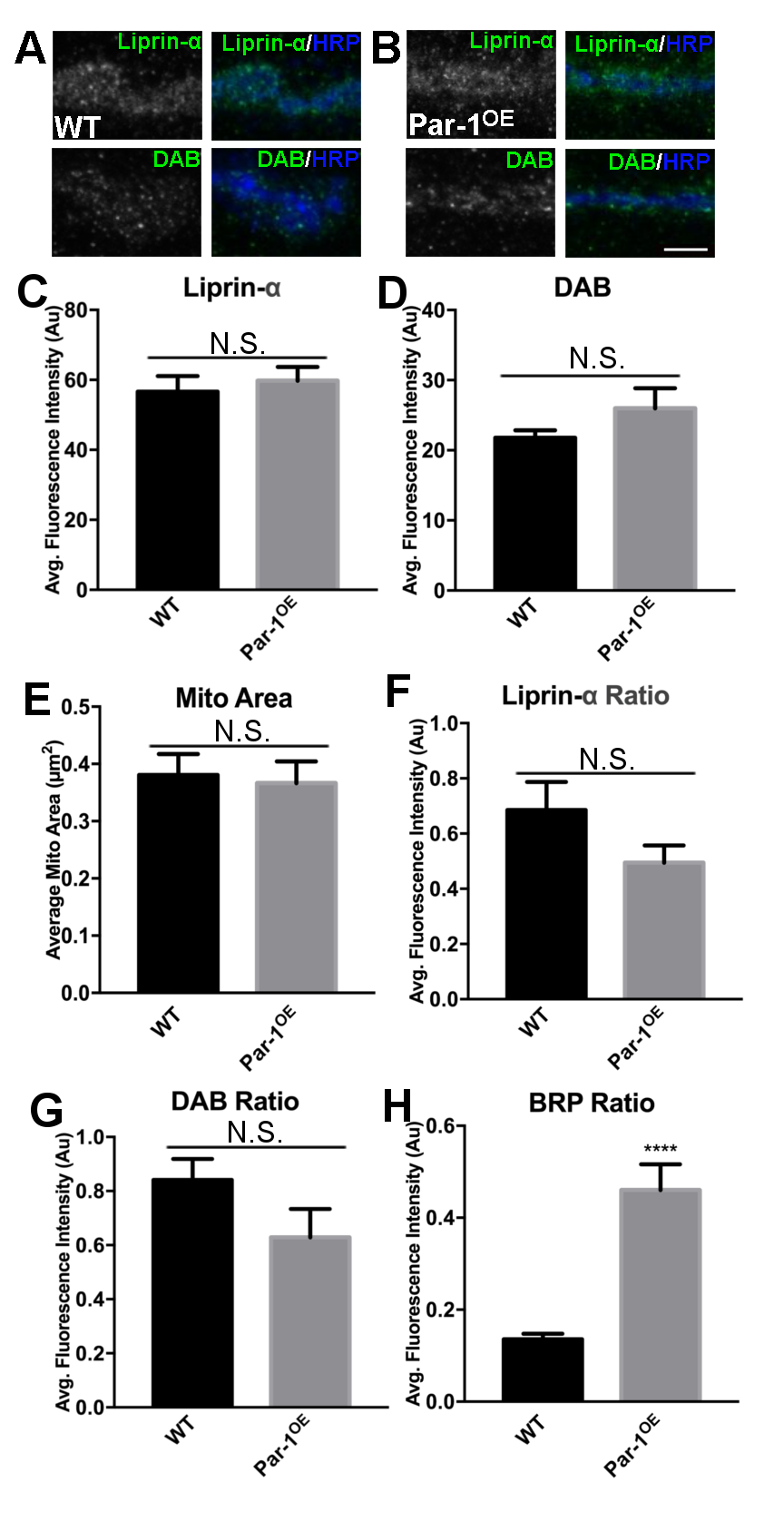

Supplement: S3 Fig — A) Representative confocal image stacks showing NMJ synapses from WT and B) Par-1OE third instar larvae stained against Liprin-α, DAB (Green) and HRP (Blue). Scale bar = 10μm.). C) Quantification of Liprin-α (Green) intensity at synapses. N = 12, p = 0.49. Error bars represent S.E.M. D) Quantification of DAB (Green) intensity at synapses. N = 12, p = 0.09. Error bars represent S.E.M. E) Quantification of Mitochondria area within axons (see Fig 2) of WT and Par-1OE larvae. N = 10, p = 0.7893. Error bars represent S.E.M. F) Quantification showing the ratio of Liprin-α intensity at axons and synapses. N = 12, p = 0.1425. Error bars represent S.E.M. G) Quantification showing the ratio of DAB intensity at axons and synapses. N = 12, p = 0.1354. Error bars represent S.E.M. H) Quantification of showing the ration of BRP intensity at axons and synapses WT and Par-1OE larvae. N = 10, **** = p<0.0001. (TIFF) [file pgen.1006621.s004.tiff]

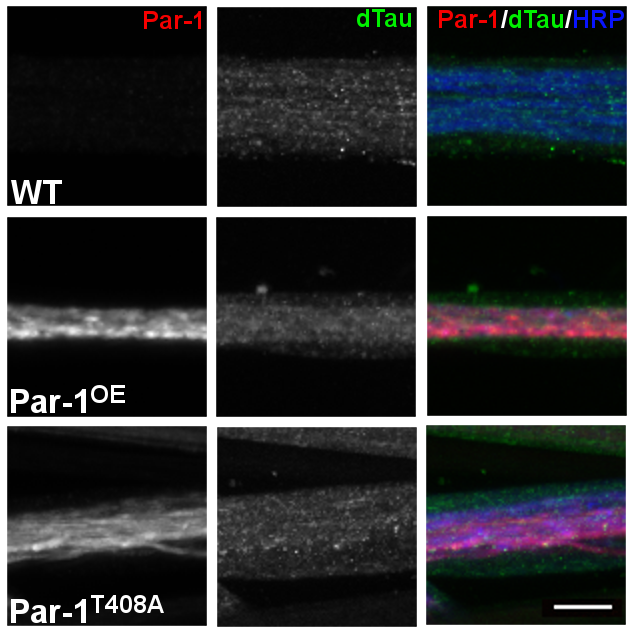

Supplement: S4 Fig — Representative images from WT and Par-1OE and Par-1T408A flies showing localization of overexpressed Par-1 (Red), endogenous tau (Green), and HRP (Blue) in axons. (TIF) [file pgen.1006621.s005.tif]

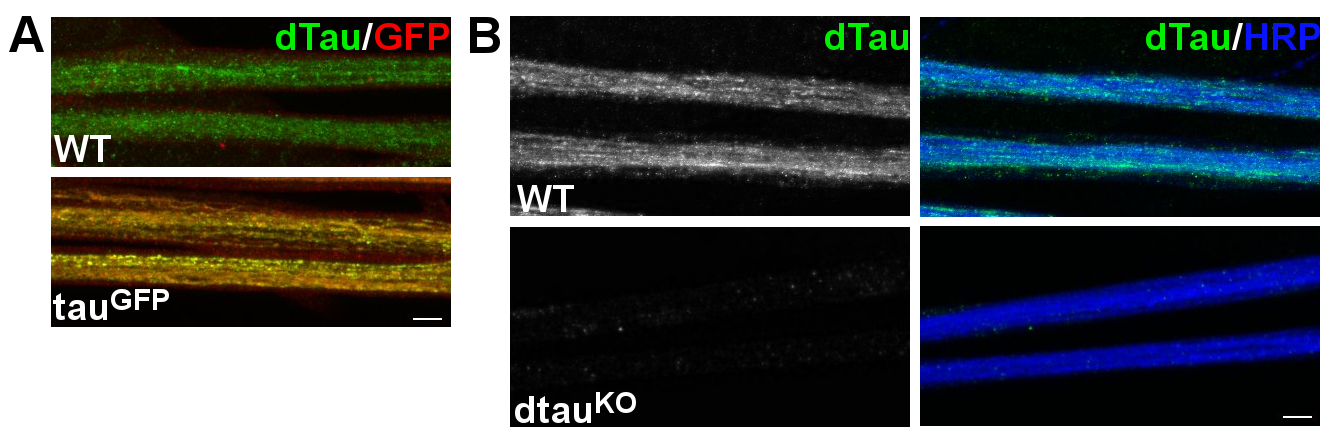

Supplement: S5 Fig — A) Representative images from WT and Par-1OE flies showing localization of overexpressed TauGFP (using anti-GFP antibody, Red) and dTau antibody (Green) in axons. Scale Bar = 10μm. B) Representative images from WT and dtauKO flies showing localization of dTau (Green) in axons and HRP (Blue). Scale Bar = 10μm. (TIFF) [file pgen.1006621.s006.tiff]

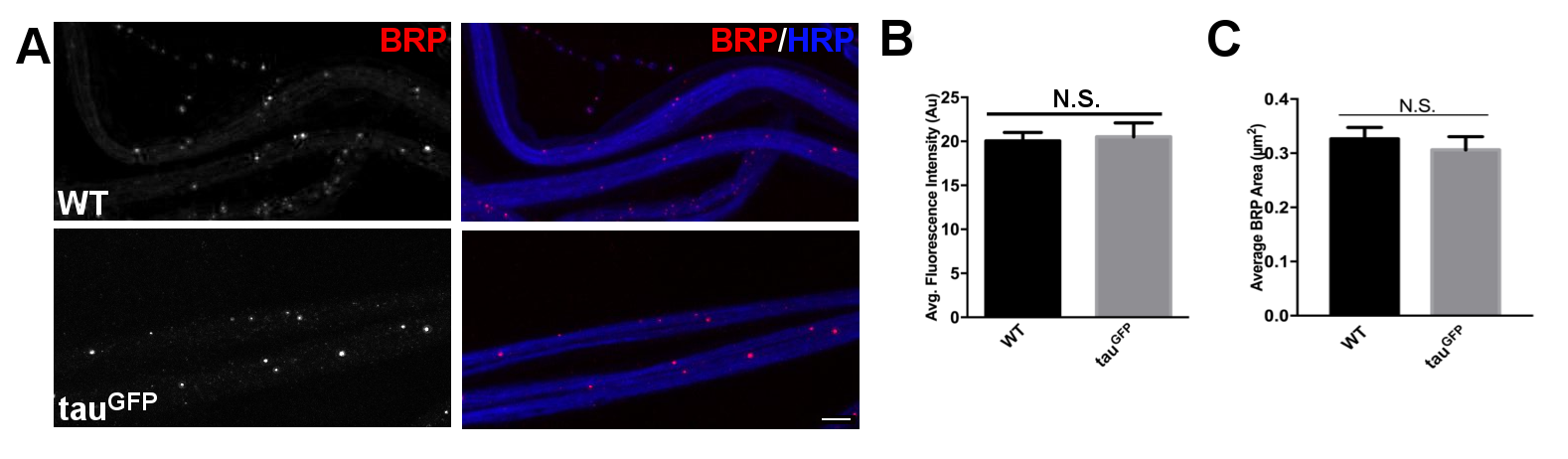

Supplement: S6 Fig — A) Representative confocal image stacks showing axons from WT and tauGFP stained with antibodies against BRP (Red) and HRP (Blue). B) Quantification of BRP intensity in axons of identical genotypes as in A. N = 10, p = 0.30. Error bars represent S.E.M. C) Quantification of BRP puncta size within axons of identical genotypes seen in A. N = 10, p = 0.52. Error bars represent S.E.M. (TIFF) [file pgen.1006621.s007.tiff]

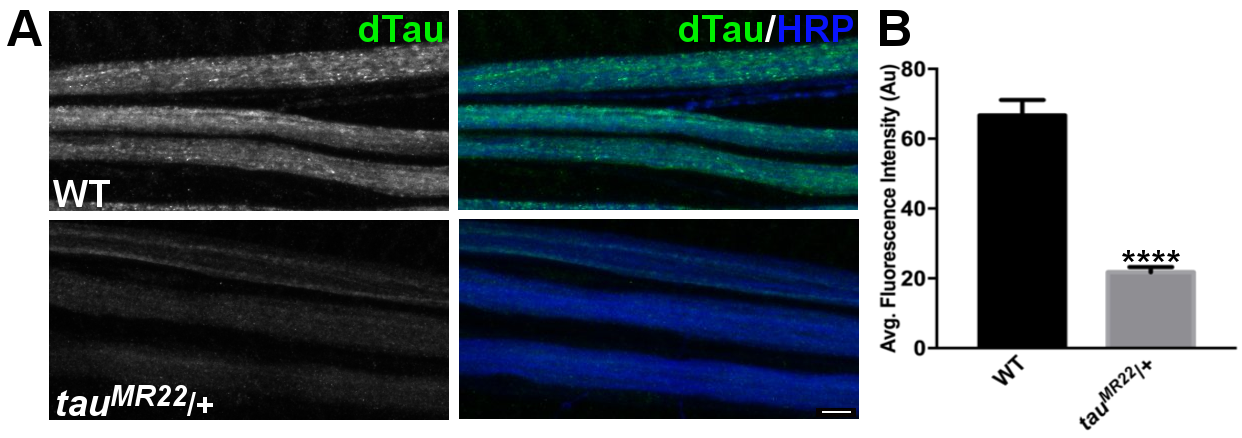

Supplement: S7 Fig — A) Representative images from WT and tauMR22/+ flies showing axons stained against dTau (Green) and HRP (Blue). B) Quantification of dTau intensity in WT and tauMR22/+ axons. N = 8, **** = p<0.0001. Scale bar = 10μm. Error bars represent S.E.M. (TIFF) [file pgen.1006621.s008.tiff]

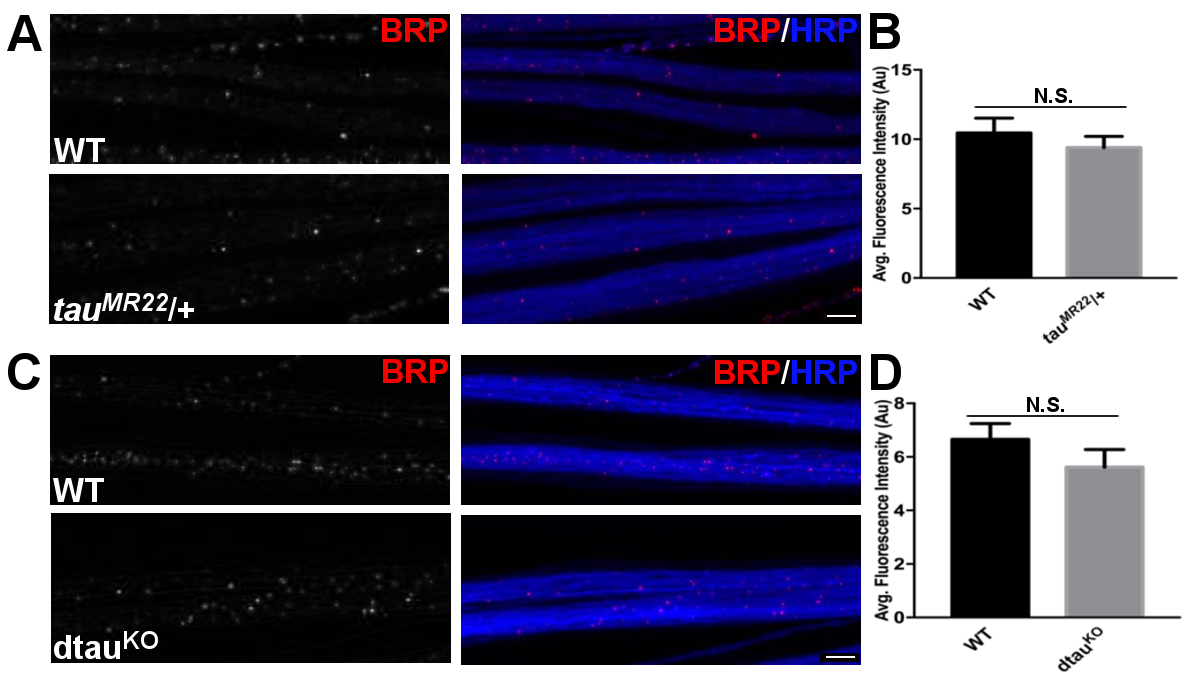

Supplement: S8 Fig — A) Representative images from WT and tauMR22/+ flies showing axons stained against BRP (Red) and HRP (Blue). B) Quantification of BRP intensity in axons. N = 8, p = 0.4309. Scale bar = 10μm. C) Representative images from WT and dtauKO flies showing axons stained against BRP (Red) and HRP (Blue). D) Quantification of BRP intensity in axons. N = 8, p = 0.24. Scale bar = 10μm. Error bars represent S.E.M. (TIFF) [file pgen.1006621.s009.tiff]

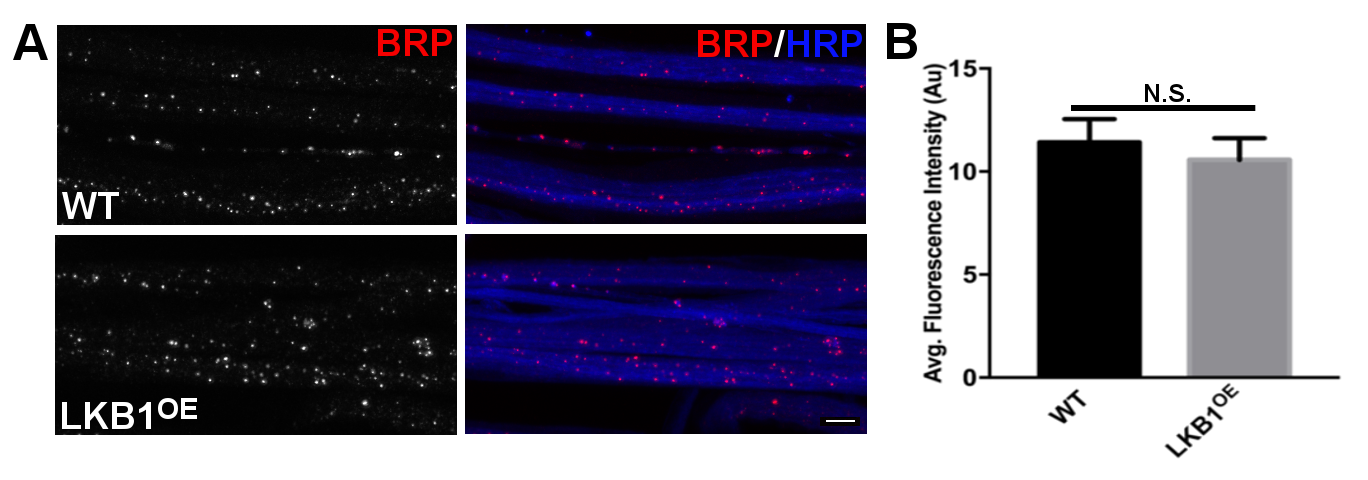

Supplement: S9 Fig — A) Representative images from WT and presynaptic overexpression of LKB1 (LKB1OE) flies driven using BG380-Gal4 showing axons stained against BRP (Red) and HRP (Blue). B) Quantification of BRP intensity in axons. N = 8, p = 0.58. Scale bar = 10μm. Error bars represent S.E.M. (TIFF) [file pgen.1006621.s010.tiff]
